# Evaluation of Fatty Acids Profile as a Useful Tool towards Valorization of By-Products of Agri-Food Industry

**DOI:** 10.3390/foods10112867

**Published:** 2021-11-19

**Authors:** Rui Ferreira, Sílvia Lourenço, André Lopes, Carlos Andrade, José S. Câmara, Paula Castilho, Rosa Perestrelo

**Affiliations:** 1 CQM—Centro de Química da Madeira, Campus da Penteada, Universidade da Madeira, 9020-105 Funchal, Portugal; rui.ferreira@staff.uma.pt (R.F.); jsc@staff.uma.pt (J.S.C.); pcastilho@staff.uma.pt (P.C.); 2 MARE—Marine and Environmental Sciences Centre, Politécnico de Leiria, Av. do Porto de Pesca, 2520-641 Peniche, Portugal; slourenco2@gmail.com; 3 CIIMAR/CIMAR—Centro Interdisciplinar de Investigação Marinha e Ambiental, Universidade do Porto, Terminal de Cruzeiros do Porto de Leixões, Av. General Norton de Matos, S/N, 4450-208 Matosinhos, Portugal; carlos.a.andrade@madeira.gov.pt; 4 OOM—Observatório Oceânico da Madeira, Edifício Madeira Tecnopolo, Piso 0, Caminho da Penteada, 9020-105 Funchal, Portugal; andre_lopes564@hotmail.com; 5 CCMAR—Centro de Ciências do Mar, Campus de Gambelas, Universidade do Algarve, 8005-139 Faro, Portugal; 6 Departamento de Química, Faculdade de Ciências Exatas e Engenharia, Campus da Penteada, Universidade da Madeira, 9020-105 Funchal, Portugal

**Keywords:** agri-food by-products, fatty acids, GC–FID, FTIR, functional quality

## Abstract

Worldwide, the food industry generates a large number of by-products from a wide variety of sources. These by-products represent an interesting and economical source of added value components with potential functionalities and/or bioactivities, which might be explored for industrial purposes, encouraging and promoting the circular economy concept. In this context, the current work aimed to evaluate the fatty acids (FAs) profile using gas chromatography–flame ionization detector (GC–FID) and Fourier Transform Infrared (FTIR), as well as the determination of related health lipid indices (e.g., atherogenic (AI) and thrombogenic (TI)) as a powerful strategy to investigate the potential applications of different agri-food by-products for human nutrition and animal feeding. This work results showed that polyunsaturated fatty acids (PUFAs) are the predominant group in grape pomace (72.7%), grape bunches (54.3%), and brewer’s spent grain (BSG, 59.0%), whereas carrot peels are dominated by monounsaturated fatty acids (MUFAs, 47.3%), and grape stems (46.2%), lees (from 50.8 to 74.1%), and potato peels (77.2%) by saturated fatty acids (SFAs). These findings represent a scientific basis for exploring the nutritional properties of agri-food by-products. Special attention should be given to grape pomace, grape bunches, and BSG since they have a high content of PUFAs (from 54.3 to 72.7%) and lower AI (from 0.11 to 0.38) and TI (from 0.30 to 0.56) indexes, suggesting their potential to provide a variety of health benefits against cardiovascular diseases including well-established hypotriglyceridemia and anti-inflammatory effects, products to which they are added.

## 1. Introduction

Food industries produce large quantities of agri-food by-products or waste, around 90 million tons per year, from which 38% of them result from food processing [1]. The agri-food by-products discarded to the environment require a high cost to waste treatment procedures and represent an additional charge to the food manufacturer. On the other hand, agri-food by-products have noteworthy nutritional value and represent an interesting source of bioactive, phytochemical (e.g., polyphenols, organic acids, fibers, polysaccharides, proteins) and technologically relevant compounds, which given their low commercial cost, constitute a suitable potential source of food additives as well as health-promoting agents for the food industry [2,3,4].

The European Union (EU) circular economy framework includes approaches to decrease the agri-food by-products through the reduction, reuse, and recycling the raw materials for the development of new products, improving the value and subsequently the useful life of raw materials and resources in the economy [4,5,6]. Moreover, the main aim of circular economy is the increment of resource efficiency by maintaining materials in usage for the lengthiest possible in technical and biological cycles. However, this would be attended with the reinforcement of natural systems and the design out of waste and pollution to guarantee the enlargement of a sustainable circular economy. The combination of circular economy codes through technological and non-technological novelties in agricultural and food manufacture and consumption systems can contribute to rising resource efficacy and decrease significantly the environmental footprint [7]. In addition, several studies have reported that the food manufacturing system could connect to the circular economy model by adjusting its production model and valorizing by-products of the agri-food industry [8].

*Vitis vinifera* L. grape is one of the main fruit crops cultivated worldwide, with an annual production of more than 67 million tons. From these, 80% of the total harvest grapes are applied in the wine-making process [5]. However, the crashing–pressing processes and the wine clarification inevitably involve generation of millions of tons of residues (e.g., grape pomace, grape stems, lees), representing a waste management issue both ecologically and economically [5,9,10]. Some of these by-products are rich in bioactive compounds such as FAs and polyphenols, which are attractive from a nutraceutical perspective [10,11,12]. Their health benefits result from antioxidant, antimicrobial, hypoglycemic, and anticarcinogenic properties [10,13,14] and, in addition, can also be used in the technological process such as colorant and texturizing agents [15,16].

A beer-brewing process similar to winemaking involves the production of millions of tons of residues, with the brewer’s spent grain (BSG) as the largest by-product (38.6 × 10^6^ t worldwide, 85% of the total by-products). BSG is essentially composed of the barley grain husks acquired as solid residue afterward the wort production [17]. BSG is a raw material with applications in food (as an ingredient and/or value-added components) and/or in non-food products (e.g., pharmaceuticals, food packaging). This by-product is a prized source of individual components (e.g., proteins, fibers, FAs) due to its high nutritional value, low cost, and high availability throughout the year [2,17,18].

FAs are crucial in living organisms since they play numerous roles, namely as source of energy, structural and modulators of physiological functions [19]. Based on their chemical structure, FAs can be organized into three classes: saturated (SFA, without double bond, CH_3_(CH_2_)_n_COOH), monounsaturated (MUFAs, with a single double bond), and polyunsaturated fatty acids (PUFAs, with two or more double bonds). They contain a hydrophilic carboxyl group attached to a carbon chain ranging from C2 (two carbon atoms) to 32 with an additional terminal methyl group [20,21]. Long-chain (LC-) PUFAs, such as eicosapentaenoic acid (EPA, 20:5n-3), docosahexaenoic acid (DHA, 22:6n-3), and arachidonic acid (ARA, 20:4n-6), are the most important providing health benefits at all life stages [20,21]. These include decreasing the risk of myocardial infarction, diabetes mellitus, and even some types of cancer [19]. Moreover, high consumption of LC-PUFAs is associated with higher cognitive performance and a lower risk of developing Alzheimer’s disease [22]. The PUFA/SFA and PUFA n-6/n-3 ratios, hypo- and hypercholesterolemic FAs (H/H), the atherogenic index (AI), and the thrombogenic index (TI) have become approximately of the utmost significant factors for assessing the nutritional value and healthiness of foods [23].

There are several extraction techniques and analytical approaches reported for the assessment of FAs in food by-products. Usually, the lipid fractions are extracted using different organic solvents and mixture of it (e.g., methanol, ether) [24,25,26], Soxhlet [27,28], and supercritical fluid extraction [28,29]. Regarding analytical approaches, infrared spectroscopy, capillary electrophoresis, liquid (LC), and gas chromatography (GC) have been reported in the determination of FAs profile [30]. From these, GC using a flame ionization detector (FID) is the most used since it is a sensitive, reproducible, accurate, and versatile approach for the analysis of complex samples that led to specific and fast analysis of FAs in a diversity of foods [25,26,31,32]. The main drawback of this analytical approach is that most FAs determined by GC–FID require derivatization due to the high boiling points of FAs, which are difficult to evaporate and have a low FID response [32]. Several derivatization procedures are reported in the literature, being the four the most common acid or base-catalyzed methylation, borontrifluoride methylation with diazomethane, and silylation [24].

Aquafeeds is a growing sector within food industry manufacturers due to the increasing importance of the aquaculture production sector for human consumption [33]. In the aquafeed industry, the search for new sources of PUFA is increasingly important due to the growing concerns with the environmental sustainability of traditional sources of EFA, plant oils, and particularly fish oil [34]. Most fish and crustaceans are unable to synthesize PUFA de novo, though some of them can convert 18-C PUFA into n-3 and n-6 long-chain PUFA, particularly ARA [35,36]. Some agriculture by-products are potential sources of 18-C PUFA, particularly linoleic acid (C18:2n6), a precursor of ARA [34].

The research behind the current study has a comprehensive scope in the perspective of a circular economy of the food industry. There are some studies related to FAs of agri-food by-products such as grape by-products (e.g., pomace, stems, bunch) [25,31] and BSG [26,27,37]. Nevertheless, few studies have been reported related to FAs in wine lees [38] and potato peels [39], and as far as we know, no studies were performed using carrot peels. Therefore, the present study aims to evaluate the FAs profile of different agri-food by-products, namely grape pomace, grape stems, grape bunches, white less, red lees, BSG, carrot, and potato peels using chromatographic and spectrometric approaches, as a strategy to evaluate its potential application for food-based matrices enrichment. A functional quality approach previously used to evaluate dietary factors involved in coronary heart disease [40] was followed to determine the most promising healthy agri-food by-products as FA sources for nutritional purposes.

## 2. Materials and Methods

### 2.1. Chemicals

Standards for gas chromatography (GC) analysis: Supelco 37 Component FAME (fatty acid methyl ester) Mix (Bellefonte, PA, USA) purity higher than 98%. Tridecanoic acid (C13:0, ˃98%), used as internal standard (IS, 10 mg/mL), and chloride acid (HCl, 37%) were purchased from ACROS Organic (Loughborough, Leicestershire, UK). Methanol (99.9%) and n-hexane (˃95%) were purchased from Sigma Aldrich (Munich, Germany).

### 2.2. Agri-Food By-Products

Grape pomaces, grape stems, grape bunches (obtained as a by-product of grape bunch pruning), and lees from red and white wine grapes were generously donated by Madeira Wine Company, whereas the brewer’s spent grains (BSG) were made available by Empresa de Cervejas da Madeira (ECM). Finally, vegetable by-products were obtained from vegetables processing plant (Dourada dos Prazeres, Funchal, Portugal). All these companies are located on Madeira Island, Portugal.

### 2.3. Extraction and Derivatization of Fatty Acids

For extraction procedure, 5 g of agri-food by-products was subjected to Soxhlet extraction for 23 h (3 cycles/h) with 350 mL of n-hexane. The n-hexane layer was concentrated on a rotary evaporator (Buchi R-114 with water bath B-480). Then, the extract obtained was lyophilized (Christ Alpha 1–2 LDplus) and submitted to transmethylation by derivatization using an alkylation reagent.

The FAs were methylated by adding 1 mL of 5% HCl in methanol, 15 µL of C13:0 (IS) to 50 mg of each lyophilized agri-food by-product extract for 20 min at 105 °C. To promote the separation of hydrophilic components, 1 mL of distillate water was added, and the FAMEs were extracted with 1 mL of n-hexane. This modified approach is cost-effective, less intrusive, and fast in terms of sample preparation [41,42]. All extractions and derivations were performed in triplicate.

### 2.4. Method Validation

Method validation was assessed in terms of selectivity, linearity, precision and sensitivity. The linearity was determined by ranging the individual FAME concentration, as presented in Table 1, and by plotting the relative area versus concentration. The intra- and inter-day precision were evaluated by replicate (*n* = 6) injection of the same standard solution containing FAMEs on the same day and three consecutive days, respectively. The results were expressed as a percentage of relative standard deviation (% RSD). The limit of detection (LOD) and limit of quantification (LOQ) were determined by the multiplications by 3 and 10 of the ratios of standard deviation(s) of calibration curve interception, by the slope of a regression curve. The accuracy was determined by spiking grape pomace with a middle-level concentration of FAMEs (10 µg/mL) and analyzed in triplicate before and after spiking.

### 2.5. Gas Chromatography–Flame Ionization Detector (GC–FID) Conditions

The FAMEs were analyzed using a certified method specific for standard solution Supelco 37 Components FAME Mix. The FAMEs separation was performed using an Agilent 7890A gas chromatograph (Agilent, Santa Clara, CA, USA) equipped with a flame ionization detector (FID) and an autosampler Agilent 7693. The analyses were carried out on a SPB^TM^-PUFA fused silica capillary column (30 m × 0.25 mm × 0.2 µm). Helium was used as carrier gas with a flow rate of 800 µL/min. The GC oven temperature started at 50 °C for 2 min, increased to 210 °C at 10 °C/min, and held for 60 min, with a total runtime of 78 min. Injector and FID detector temperatures were held at 250 and 260 °C, respectively. The injection volume was 1 µL using a split injection with a split ratio (volume of gas passing down the capillary column) of 100:1, with a solvent delay time of 3.7 min. Air and hydrogen were supplied to the FID detector at flow rates of 450 and 40 mL/min, respectively. All analyses were done in triplicate.

### 2.6. Functional Quality

The functional quality of agri-food by-products was assessed through the ratio between hypo- and hypercholesterolemic FAs (H/H), the atherogenic index (AI), and the thrombogenic index (TI), calculated according to equations described by Ulbricht and Southgate [40].

H/H = (C18:1 + C18:2 + C18:3)/(C14:0 + C16:0)
AI = (C12:0 + 4 × C14:0 + C16:0)/[Σ MUFA + Σ PUFA (n6 & n3)]
TI = (C14:0 + C16:0 + C18:0)/[0.5 × Σ MUFA + 0.5 × Σ PUFA (n6) + 3 × Σ PUFA (n3) + [Σ PUFA (n3)/Σ PUFA (n6)]

where C14:0—myristic acid, C16:0—palmitic acid, C18:0—stearic acid, C18:1—oleic acid, C18:2—linoleic acid, C18:3—α-linolenic acid, Σ MUFA—the sum of monounsaturated FAs, Σ PUFA (n3)—the sum of the polyunsaturated n3 FAs, and Σ PUFA (n6)—the sum of the polyunsaturated n6 FAs.

The FAs profile could be a valuable indicator for the evaluation of functional qualities of agri-food by-products. The unsaturation ratio (U/S) is often used to determine indexes for recurrent cardiovascular disease syndromes (e.g., atherogenicity, thrombogenicity) since only three SFAs are hypercholesterolemic [40,43].

### 2.7. Fourier Transform Infrared Spectroscopy (FTIR) Analysis of Agri-Food By-Products

The FTIR spectra were recorded on a Perkin Elmer Spectrum two spectrometer (Perkin Elmer, Waltham, MA, USA) combined to an ultra-attenuated total reflectance (UATR) two, diamond crystal, single reflection. The data acquisition was carried out by IR SPECTRUM^TM^ 10.6 software package (Perkin Elmer, Waltham, MA, USA). The spectra were acquired (32 scans per background or food by-product) in the range of 4000–400 cm^−1^ at a nominal resolution of 4 cm^−1^. The spectra baseline was normalized using the background spectrum of air. For each analysis, a lyophilized sample (~10 mg) was put on the surface of the ATR crystal. Each agri-food by-product was analyzed in triplicate to evaluate the method’s reproducibility.

### 2.8. Statistical Analysis

All assays were carried out in triplicate to guarantee statistical significance. The data were analyzed by one-way analysis of variance (ANOVA) followed by Tukey’s multiple comparison test using MetaboAnalyst 5.0 software developed by the University of Alberta, Canada [44]. The value *p* < 0.05 was taken as statistically significant.

## 3. Results and Discussion

### 3.1. Method Validation

Quantitative analysis of FAMEs was carried out by assessing the performance of the analytical method through the assessment of selectivity, linearity, precision (intra- and inter-days), sensitivity (LOD and LOQ), and accuracy. Table 1 shows the figures merit for FAMEs detected in the agri-food by-products.

The selectivity of the method was evaluated by the nonappearance of interfering peaks at the retention time (RT) of studied FAMEs, as evidenced by Figure S1 (Supplementary Material). The linearity of the method was measured through calibration curves that were fit using least square linear regression analysis. The obtained correlation coefficient (R^2^) was higher than 0.981 with residuals not exceeding ±15%, showing the method linearity over the whole range of concentration considered for most of the studied FAMEs. The LOD value ranged from 0.01 (C10:0) to 0.41 (C4:0) µg/mL, whereas the LOQ ranged from 0.03 (C10:0) to 1.38 (C4:0) µg/mL, showing the method is sensitive to quantify FAMEs in the matrices investigated. The obtained intra-day precision, expressed as % relative standard deviation (%RSD), for all FAMEs ranged from 1.01 to 4.89%, whereas the inter-day precision from 2.64 to 12.9%. For the grape pomace spiked with a middle concentration of FAMEs standard (10 µg/mL), the recovery ranged from 80.3 to 118.2%. The good recoveries attained in the current study indicated that the derivatization procedure used in the determination of FAs in agri-food by-products was suitable.

### 3.2. Lipid Extraction Yield and Fatty Acids Profile and Functional Quality of Agri-Food By-Products

The current research aims the development of a valorization strategy for agri-food by-products, and for this purpose, Soxhlet n-hexane extraction was performed to obtain the lipid fraction. The lipid extracted (% *w/w* of dried agri-food by-product) was the highest for grape bunches (11.2% *w/w*), followed by BSG (8.58% *w/w*), red lees (6.46% *w/w*), white less (3.41% *w/w*), grape stems (4.28% *w/w*), grape pomace (3.18% *w/w*), carrot peel (1.45% *w/w*), and potato peel (0.70% *w/w*). This research was conducted at a laboratory scale; however, for an industrial purpose, green extraction (e.g., supercritical carbon dioxide extraction scCO_2_) should be considered to obtain lipid extraction of agri-food by-products. scCO_2_ compared to Soxhlet presents several advantages such as shorter extraction times, low environmental impact, and non-toxicity, which makes it suitable for the industry application and guarantees clean extracted products. This fact does not represent a concern in future applications since several studies have already demonstrated that the lipid extraction yield obtained by Soxhlet and scCO_2_ was quite similar [28,29,45]. Nevertheless, at the laboratory scale, the Soxhlet extraction procedure remains the most available to be used for this purpose and obtain preliminary chemical information about the extracts.

The qualitative analysis was carried out by assessing the GC–FID chromatograms of all agri-food by-products (Figures S2–S9) and comparing the RT of FAMEs with the standards, using the same chromatographic conditions. Table 2 summarizes the qualitative and quantitative information of FAMEs profile of agri-food by-products. Related to quantitative data, the results were expressed as g per 100 g of dried sample. 

Regarding qualitative analysis, 5 FAMEs were identified in grape bunches, 6 in BSG, 7 in grape pomace, 8 in white lees, 9 in potato peels, 10 in grape stems and in red lees, and 18 in carrot peels. Only five FAMEs were common to all agri-food by-products, namely methyl palmitate (C16:0), methyl stearate (C18:0), methyl oleate (C18:1n9), methyl linoleate (C18:2n6), and methyl α-linolenate (C18:3n3). On the other hand, methyl butanoate (C4:0) was only identified in potato peels, and methyl octanoate (C8:0), methyl pentadecanoate (C15:0), methyl pentadecenoate (C15:1n5), and methyl 11-eicosatrienoate (C20:1n9) were only identified in carrot peels.

Methyl linoleate (C18:2n6) is the most predominant FAME on the investigated grape by-products and BSG, and its content varied from 25.1% (grape stems) to 70.8% (grape pomace). No significant difference at *p* < 0.05 was observed in methyl linoleate (C18:2n6) content between grape bunches (52.0%) and BSG (53.9%), Table 2. Linoleic acid is an essential PUFA (n-6) that acts as a structural component of membranes and as precursors of eicosanoids, which modulate pulmonary and renal functions. In fish, linoleic acid is associated with the production of eggs yolk, suggesting an important role during embryonic and larval development [35]. In addition, it is used in pharmaceutical and cosmetic formulations, endorsing the activity of vitamins A and E, as well as acting as barrier properties after stratum corneum recovery [31]. On the other hand, methyl dodecanoate (C12:0, 18.5%) and methyl linoleate (C18:2n6, 18.3%) were the most abundant FAMEs in white lees. Their content in wine lees did not differ significantly at *p* < 0.05. In addition, no significant difference was observed in the content of methyl linoleate (C18:2n6) between white and red lees. Methyl palmitate (C16:0) was the most abundant FAME in red lees (32.3%). The consumption of high levels of SFAs in specific palmitic acid (C16:0) causes inflammatory responses, which are an important factor in the development of diseases associated with obesity, and insulin resistance [46]. In carrot peels, the most abundant FAME was methyl oleate (C18:1n9), whereas in potato peels, it was methyl lignocerate (C24:0).

The total content of SFAs ranged from 15.3% (grape pomace) to 77.2% (potato peels). Nevertheless, no significant difference was observed between the total content of SFAs between grape pomace (15.3%) and grape bunches (18.2%), as well as between potato peels (77.2%) and white lees (74.1%). Methyl hexadecanoate (C16:0) was the most abundant SFA in all agri-food by-products, except in white lees. No significant difference was observed in the content of methyl hexadecanoate (C16:0) among grape bunches (14.3%), white lees (11.3%), and carrot peels (13.2%). The total content of MUFAs ranged from 3.88% (white lees) to 47.3% (carrot peels). No significant difference was observed in the total content of MUFAs between white lees (3.88%) and potato peels (5.06%). Methyl oleate (C18:1n9) was the only MUFA identified in grape bunches (27.4%), white lees (3.88%), BSG (11.7%), and in potato peels (5.06%). The total content of PUFAs ranged from 17.0% (carrot peels) to 72.7% (grape pomace). No significant difference was observed in the total content of PUFAs between carrot (17.0%) and potato peels (17.8%), as well as between white (22.0%) and red lees (22.2%).

Previous studies have indicated that the grape pomace and bunches are a rich source of PUFAs (69–75%, represented mainly by linoleic acid) and poor in MUFAs (14–19%) and SFAs (11–12%) [25,31,43,47]. On the other hand, grape stems are composed of high content of SFAs (24–31%) and α-linoleic acid (10–15%) [25,31,43,47]. Our results agree with these studies. BSG showed the highest content of PUFAs (59.0%, being methyl linoleate the most predominant), followed by SFAs (29.3%, methyl palmitate), and finally by MUFAs (11.7%, methyl oleate), as reported in previous studies [26,27]. BSG represents an interesting source of FAs, with relevant use by the food industry as functional food ingredients since it is related to cholesterol lowering [26]. In addition, white and red lees showed a high content of SFAs (from 50.8 to 74.1%), which are in agreement with a study performed by Sancho-Galán et al. [38].

**Figure 1:**
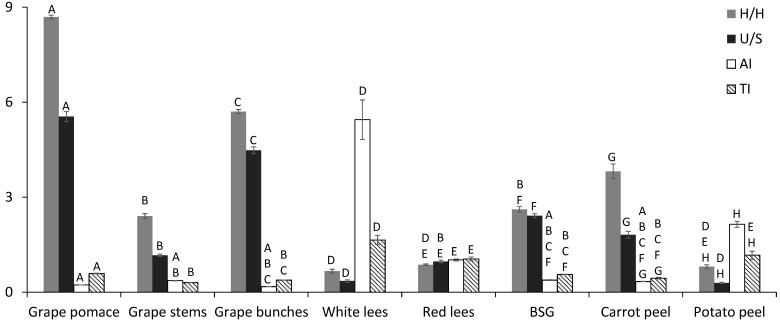
Hypo- and hypercholesterolemic FAs ratio (H/H), unsaturation ratio (U/S), atherogenicity (AI), and thrombogenicity (TI) indexes of agri-food by-products. Different letters in a bar represent statistically significant differences among agri-food by-products by one-way ANOVA and Tukey’s multiple test at *p* < 0.05.

Figure 1 shows the profile of a hypo- and hypercholesterolemic FAs ratio (H/H), unsaturation ratio (U/S), atherogenicity index (AI), and thrombogenicity index (TI). The U/S indicates the proportion of unsaturated related to saturated FAs. The higher AI and TI indicate a higher risk of atherogenicity and thrombogenicity of the dietary fat. These indexes are valuable indicators of the potential effect of atherogenicity, thrombogenicity, and cardiovascular health [19]. The H/H values ranged from 0.67 (white lees) to 8.69 (grape pomace). No significant difference was observed between grape stems (2.41) and BSG (2.62) and between white (0.67) and red lees (0.87). A higher level of this index is suitable for nutrition since it expresses the influence of the FAs on cholesterol metabolism [43]. The H/H values obtained for all agri-food by-products are lower than the reported in the literature for linseed oils (13.24), sesame (7.72), and olive oils (6.14) [43], with the exception of grape pomace (8.69) that present H/H values higher than sesame and olive oils.

The U/S ratio ranged from 0.30 (potato peels) to 5.55 (grape pomace). No significant difference was observed between grape stems (1.17) and red lees (0.97) and between white lees (0.35) and potato peels (0.30). The PUFA n-6/PUFA n-3 ratio ranged from 1.44 (grape stems to 37.3 (grape pomace). As can be observed in Table 2, no significant difference in PUFA n-6/PUFA n-3 ratio was observed among grape stems (1.44), red lees (2.72), and potato peels (1.72). The U/S and PUFA n-6/PUFA n-3 ratio are commonly used to evaluate the nutrition value of fat, and according to Ahmed et al. [48], a low U/S index and a high PUFA n-6/PUFA n-3 ratio are considered unfavorable since they may induce an increase in cholesterolemia. In addition, according to Simopoulos et al. [49], an increase in PUFA n-6/PUFA n-3 ratio increases the risk of obesity.

The AI values ranged from 0.11 (grape pomace) to 5.45 (white lees), whereas the TI values ranged from 0.30 (grape pomace) to 1.65 (white lees). Regarding the AI index, no significant difference was observed between grape by-products under study. The values of AI and TI obtained in all agri-food by-products were higher than the ones reported in the literature for linseed, sesame, and olive oils [43]. Regarding the AI index, no significant difference was observed between grape by-products under study. In addition, high values of AI and TI indexes do not contribute positively to cardiovascular health. In this sense, grape pomace, grape bunches, and BSG showed the lowest AI and TI values and highest H/H and U/S ratio; consequently, they are expected to contribute positively to the improvement of cardiovascular health. The highest level of H/H and U/S ratio is desirable in nutrition since it expresses the effect of the FAs on cholesterol metabolism.

### 3.3. Fatty Acids Profile by Fourier Transform Infrared Spectroscopy (FTIR)

**Figure 2:**
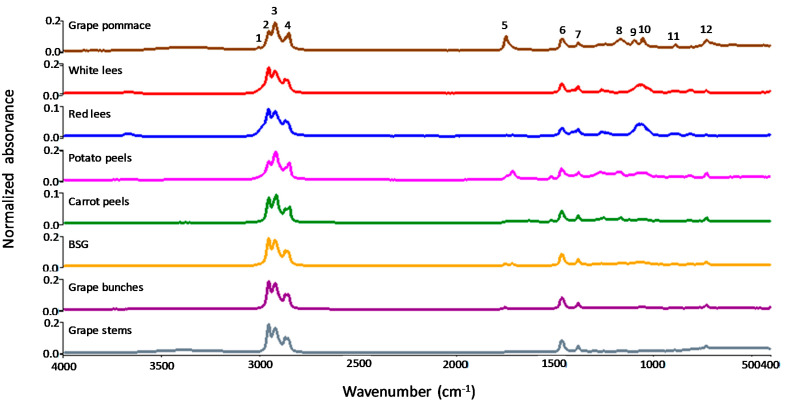
FTIR spectra of agri-food by-products in the mid-infrared region (4000–400 cm^−1^). The identification of numbers was in Table 3.

FTIR offers a typical fingerprint of the chemical components present in the sample by featuring their molecular vibrations (e.g., stretching, bending) [10]. In addition, FTIR is a cheap and fast technique that requires a very small amount of a sample and is used mainly to evaluate eventual oxidation during extraction and storage, as well as the ratio of MUFA: PUFA. Figure 2 shows the FTIR spectrum obtained for the agri-food by-products under study. The FTIR approach applied in the current study is a non-destructive technique that provides a fast and easy insight into the molecular structure and relative qualification of ω-3 FA using very small amounts of sample and little sample preparation and also can be used mainly to evaluate the eventual oxidation during extraction and storage as well as ratios of MUFA: PUFA, therefore allowing a qualitative characterization in correlation with the quantitative characterization by chromatographic approach.

Apart from grape stems, the spectra of oil samples did not show lipid autooxidation products due to the absence of bands between the wavenumber 3500–3200 cm^−1^. The IR spectra are very similar in terms of band positions and intensity, with the notable exception of grape pomace and potato peels, showing a characteristic band 5 attributed to C=O from ester-FA linkage in triglycerides at 1748 cm^−1^. The small band 1 at 3014 cm^−1^, which is characteristic of the cis-double-bond stretching of unsaturated fats, is most prominent in grape pomace (Table 3). This observation has a direct correlation with GC analysis that shows the prevalence of PUFA in the triglyceride mixtures and the more favorable ratio PUFA/SFA of this sample. This observation has a direct correlation with GC analysis that refers to the prevalence of PUFA in the triglyceride mixtures and the more favorable ratio PUFA/SFA for this sample. The strong absorption bands 2 and 3 at 2950–2925 cm^−1^ and band 4 at 2856 cm^−1^ are associated with asymmetrical and symmetrical stretching modes of the methylene groups of the fatty acid backbone, respectively. Band 6 at 1458 cm^−1^ is related to the C-H bending (scissoring) vibrations of methyl and methylene aliphatic groups, and band 7 at 1378 cm^−1^ is associated with symmetrical bending of the methyl group, whereas band 8 at 1163 cm^−1^ could be assigned to the stretching vibrations of the C-O group. The unique systematic variation in the 1100–1050 cm^−1^ region, most defined in grape pomace, white lees, and red lees, is designated the fingerprint region as previously reported by Shiroma and Rodriguez-Saona [50] and is known to be associated with C-O and C-C stretches in sugars, esters, and organic acids amongst other compounds. Band 10 is associated with the stretching vibration of acyl (C–O) ester groups. Further band assignment was done according to the literature [51].

In summary, BSG, grape pomace, and bunches were the most promising agri-food by-products for an industrial application since they presented the highest lipid yield extraction, PUFAs content, and a positive impact on cardiovascular health (lowest AI and TI values and highest H/H and U/S ratio). Moreover, from these three, BSG is the most promising since this by-product has high nutritional value, low cost, and high availability throughout the year. In contrast, potato peel and red lees were less promising for an industrial application due to their highest SFA content, AI, and TI values and lowest H/H and U/S ratio.

## 4. Conclusions

The current research work explores the potential of agri-food by-products for human nutrition and animal feeding, expressed by the FAs profile using chromatographic (GC–FID) and spectrometric (FTIR) approaches. In the current research, Soxhlet hexane extraction was performed to obtain the lipid fraction, but for an industrial application, green extraction like as scCO_2_ should be considered since it is non-toxic, requires lower extraction times, and has low environmental impacts. This does not represent a concern since previous studies have been demonstrated that lipid yield extraction obtained by Soxhlet is quite similar to scCO_2_.

A total of 19 FAMEs were identified, 11 saturated (SFAs), 6 monounsaturated (MUFAs), and 2 polyunsaturated fatty acids (PUFAs). From a nutritional point of view, grape pomace (72.7%), grape stems (42.5%), grape bunches (54.3%), and BSG (59.0%) were the most interesting agri-food by-products due to their high amount of PUFAs. On the other hand, white lees (74.1%), red lees (50.8%), and potato peels (77.2%) were the less interesting by-products due to their high amount of SFAs. High H/H ratio and low indexes values of AI and TI were observed in grape pomace, BSG, grape bunches, carrot peels, and grape stems, suggesting their potential use for the prevention of atherosclerosis, thrombosis, and cardiovascular diseases. Moreover, BSG is the most promising for an industrial application among agri-food by-products studied since this by-product has high availability throughout the year, contrary to grape pomace, grape stems, among others.

In addition, our findings represent a remarkable input for the circular economy. It is suggested that the investigated agri-food by-products can be used as a source of valuable FAs, contributing to valorizing these wastes from food industries. This appeals for additional research on the extraction, isolation, and purification of FAs from agri-food by-products for food and feed safety assurance based on the market requirements. Additionally, studies are needed on life cycle analysis concerning full economic costing of the use of agri-food by-products as human food and/or a feed ingredient.

## Figures and Tables

**Table 1:** Parameters of method validation.

FAMEs	RT (min)	R^2^	Range (µg/mL)	Equation	LOD (µg/mL)	LOQ (µg/mL)	Precision (%RSD)	
							Intra-Day	Inter-Day
C4:0	4.25	0.993	1.77–39.1	y = 0.069x + 0.354	0.41	1.38	1.88	5.02
C8:0	11.0	0.982	1.19–39.3	y = 0.119x + 1.472	0.20	0.66	1.47	8.66
C10:0	13.9	0.993	1.19–39.8	y = 0.136x + 0.708	0.01	0.03	1.15	10.2
C12:0	16.5	0.993	1.19–39.5	y = 0.136x + 0.870	0.02	0.07	1.01	2.64
C14:0	18.9	0.999	1.19–39.6	y = 0.151x + 1.112	0.22	0.74	1.32	3.73
C14:1n5	19.3	0.988	1.76–19.5	y = 0.156x + 0.155	0.19	0.63	2.56	12.6
C15:0	20.3	0.998	1.76–19.6	y = 0.158x + 0.756	0.14	0.48	1.52	10.4
C15:1n5	20.7	0.994	1.80–20.0	y = 0.139x + 0.838	0.40	1.36	1.84	7.79
C16:0	21.8	0.995	0.60–60.3	y = 0.201x + 1.395	0.12	0.40	3.09	5.35
C16:1n7	22.2	0.995	0.62–20.5	y = 0.146x + 1.003	0.07	0.24	2.76	4.17
C18:0	26.3	0.996	1.19–39.6	y = 0.234x + 0.846	0.35	1.15	3.25	5.96
C18:1n9	26.7	0.996	1.19–39.6	y = 0.379x + 1.176	0.33	1.10	4.89	7.28
C18:2n6	27.9	0.993	0.58–19.9	y = 0.302x + 0.9574	0.06	0.21	1.69	2.98
C18:3n3	29.9	0.983	1.73–19.2	y = 0.254x + 0.270	0.28	0.92	2.14	3.65
C20:0	34.1	0.981	1.19–39.6	y = 0.182x + 0.901	0.16	0.53	1.62	5.11
C20:1n9	34.8	0.992	1.79–19.9	y = 0.185x + 0.813	0.25	0.82	1.37	12.9
C22:0	48.2	0.992	1.18–39.4	y = 0.181x + 0.619	0.10	0.33	1.04	7.02
C22:1n9	49.6	0.999	0.60–20.0	y = 0.112x + 0.991	0.17	0.56	2.42	5.02
C24:0	74.1	0.990	2.52–39.1	y = 0.183x + 0.445	0.64	2.13	1.98	9.73

Mean of three replications; RT—retention time; R^2^—correlation coefficient; LOD—limit of detection; LOQ—limit of quantification.

**Table 2:** Fatty acids methyl esters (FAMEs) profile (g/100 g of dried sample ± standard deviation) of agri-food by-products.

FAMEs	Grape Pomace	Grape Stems	Grape Bunches	White Lees	Red Lees	BSG	Carrot Peel	Potato Peel
**Saturated fatty acids**								
Methyl butanoate C4:0)	-	-	-	-	-	-	-	4.67 ± 0.43
Methyl octanoate (C8:0)	-	-	-	-	-	-	2.62 ± 0.03	-
Methyl decanoate (C10:0)	-	2.43 ± 0.31 ^A^	-	15.3 ± 0.96 ^B^	-	-	2.03 ± 0.16 ^A^	-
Methyl dodecanoate (C12:0)	-	-	-	18.5 ± 1.09 ^A^	5.64 ± 0.92 ^B^	-	2.77 ± 0.12 ^C^	5.51 ± 0.66 ^B,D^
Methyl tetradecanoate (C14:0)	-	-	-	27.6 ± 1.01 ^A^	3.10 ± 0.33 ^B^	-	1.43 ± 0.17 ^C^	5.02 ± 0.46 ^D^
Methyl pentadecanoate (C15:0)	-	-	-	-	-	-	1.33 ± 0.16	-
Methyl hexadecanoate (C16:0)	9.69 ± 0.13 ^A^	19.7 ± 0.67 ^B^	14.3 ± 0.30 ^C^	11.3 ± 1.18 ^A,C,D^	32.3 ± 0.31 ^E^	27.0 ± 0.66 ^F^	13.2 ± 0.69 ^C,D,G^	23.3 ± 1.36 ^H^
Methyl stearate (C18:0)	4.57 ± 0.34 ^A^	4.52 ± 0.06 ^A,B^	3.90 ± 0.57 ^A,B,C^	1.38 ± 0.10 ^D^	9.77 ± 0.59 ^E^	1.59 ± 0.24 ^D,F^	2.79 ± 0.17 ^G^	8.50 ± 0.35 ^H^
Methyl eicosanoate (C20:0)	1.04 ± 0.18 ^A^	2.91 ±0.14 ^B^	-	-	-	0.69 ± 0.17 ^A,C^	3.02 ± 0.30 ^B,D^	-
Methyl behenate (C22:0)	-	7.61 ± 0.82 ^A^	-	-	-	-	3.39 ± 0.20 ^B^	-
Methyl lignocerate (C24:0)	-	9.02 ± 0.79 ^A^	-	-	-	-	2.90 ± 0.15 ^B^	30.2 ± 0.71 ^C^
**Monounsaturated fatty acids**								
Methyl 9-tetradecenoate (C14:1n5)	-	-	-	-	3.16 ± 0.59 ^A^	-	1.65 ± 0.30 ^B^	-
Methyl pentadecenoate (C15:1n5)	-	-	-	-	-	-	1.28 ± 0.06	-
Methyl palmitoleate (C16:1n7)	0.57 ± 0.07 ^A^	-	-	-	4.90 ± 0.61 ^B^	-	2.25 ± 0.30 ^C^	-
Methyl oleate (C18:1n9)	11.5 ± 1.85 ^A^	4.82 ± 0.24 ^B^	27.4 ± 0.38 ^C^	3.88 ± 0.58 ^B,D^	8.44 ± 0.43 ^E^	11.7 ± 0.12 ^A,F^	38.6 ± 0.85 ^G^	5.06 ± 0.43 ^B,D,H^
Methyl 11-eicosatrienoate (C20:1n9)	-	-	-	-	-	-	2.00 ± 0.05	-
Methyl erucate (C22:1n9)	-	6.53 ± 0.73 ^A^	-	-	10.7 ± 0.07 ^B^	-	1.44 ± 0.04 ^C^	-
**Polyunsaturated fatty acids**								
Methyl linoleate (C18:2n6)	70.8 ± 1.53 ^A^	25.1 ± 0.39 ^B^	52.0 ± 0.48 ^C^	18.3 ± 1.47 ^D^	16.2 ± 0.59 ^D,E^	53.9 ± 0.89 ^C,F^	14.7 ± 0.51 ^E,G^	11.2 ± 0.03 ^H^
Methyl α-linolenate (C18:3n3)	1.94 ± 0.32 ^A^	17.4 ± 0.42 ^B^	2.29 ± 0.39 ^A,C^	3.80 ± 0.28 ^D^	6.00 ± 0.63 ^E^	5.17 ± 0.42 ^E,F^	2.28 ± 0.05 ^A,C,G^	6.58 ± 0.80 ^E,H^
Σ SFA	15.3 ^A^	46.2 ^B^	18.2 ^A,C^	74.1 ^D^	50.8 ^E^	29.3 ^F^	35.5 ^G^	77.2 ^D,H^
Σ MUFA	12.1 ^A^	11.3 ^A,B^	27.4 ^C^	3.88 ^D^	27.2 ^C,E^	11.7 ^A,B,F^	47.3 ^G^	5.06 ^D,H^
Σ PUFA	72.7 ^A^	42.5 ^B^	54.3 ^C^	22.0 ^D^	22.2 ^D,E^	59.0 ^F^	17.0 ^G^	17.8 ^G,H^
PUFA n3/PUFA n6	0.03 ^A^	0.70 ^B^	0.04 ^A,C^	0.21 ^D^	0.37 ^E^	0.10 ^A,C,F^	0.16 ^D,F,G^	0.59 ^H^
PUFA n6/PUFA n3	37.3 ^A^	1.44 ^B^	23.2 ^C^	4.81 ^B,D^	2.72 ^B,D,E^	10.5 ^D,E,F^	6.42 ^D,E,F,G^	1.72 ^B,D,E,G,H^

Mean of three replications; SFA—saturated fatty acids; MUFA—monosaturated fatty acids; PUFA—polyunsaturated fatty acids; BSG—brewer’s spent grain. Different letters in a row represent statistically significant difference among agri-food by-products by one-way ANOVA and Tukey’s multiple test at *p* < 0.05.

**Table 3:** Assignment of the infrared (IR) band.

Band	Frequency (cm^−1^)	Functional Group	Mode of Vibration
1	3014	CH=CH (cis)	Stretching
2	2953	R-CH_3_ (methyl)	Stretching (asym)
3	2925	R=CH_2_ (methylene)	Stretching (asym)
4	2856	R=CH_2_	Stretching (sym)
5	1748	C=O (ester-FA linkage)	Stretching
6	1458	R=CH_2_	Bending (scissoring)
7	1378	R-CH_3_	Bending (sym)
8	1163	R =CH-R	Stretching
9–10	1100–1050	C-O (ester)	Stretching (asym)
11	880	R=CH (cis or trans)	Bending out of plate
12	725	R=CH_2_ or R-(CH_2_)_n_	Rocking (overlapping)

Asym—asymmetric; Sym—symmetric.

## Data Availability

Not applicable.

## Supplementary Materials

The following are available online at https://www.mdpi.com/article/10.3390/foods10112867/s1, Figure S1: GC-FID chromatograms of fatty acids methyl esters (FAMEs) standards; Figure S2: GC-FID chromatograms of fatty acids methyl esters (FAMEs) identified in grape pomace; Figure S3: GC-FID chromatograms of fatty acids methyl esters (FAMEs) identified in grape stems; Figure S4: GC-FID chromatograms of fatty acids methyl esters (FAMEs) identified in grape bunches; Figure S5: GC-FID chromatograms of fatty acids methyl esters (FAMEs) identified in white lees; Figure S6: GC-FID chromatograms of fatty acids methyl esters (FAMEs) identified in red lees; Figure S7: GC-FID chromatograms of fatty acids methyl esters (FAMEs) identified in BSG; Figure S8: GC-FID chromatograms of fatty acids methyl esters (FAMEs) identified in carrot peel; Figure S9: GC-FID chromatograms of fatty acids methyl esters (FAMEs) identified in potato peel.
