# Supplementary material for: Evaluation of Fatty Acids Profile as a Useful Tool towards Valorization of By-Products of Agri-Food Industry
Source: Foods. 2021 Nov 19;10(11):2867. doi: 10.3390/foods10112867 (PMC8624466; doi:10.3390/foods10112867)
Supplement: Supplementary file 1 [file foods-10-02867-s001.zip › foods-1458088-Supplementary Materials.pdf]

foods MDPI

Supplementary Materials

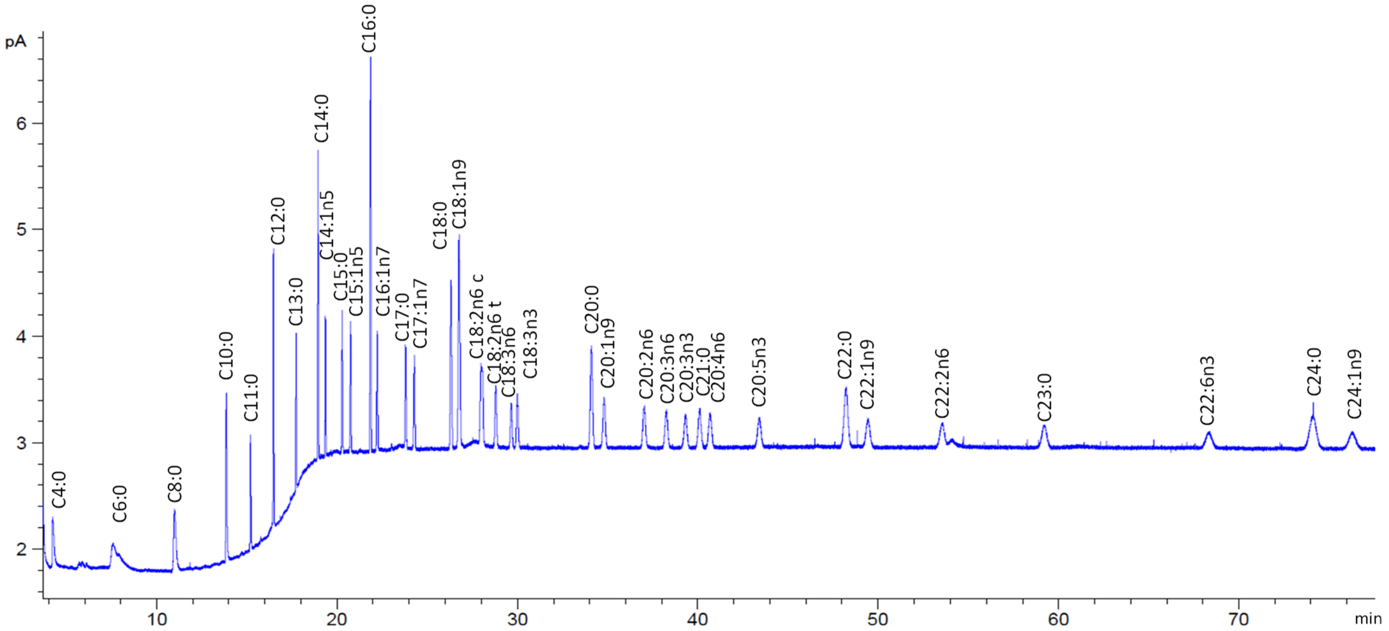

Figure S1. GC-FID chromatograms of fatty acids methyl esters (FAMEs) standards. Peak identification: C4:0 - methyl butanoate; C6:0 - methyl hexanoate; C8:0 - methyl octanoate; C10:0 - methyl decanoate; C11:0 - methyl undecanoate; C12:0 - methyl dodecanoate; C13:0 - methyl tridecanoate; C14:0 - methyl tetradecanoate; C14:1n5 - methyl 9-tetradecenoate; C15:0 - methyl pentadecanoate; C15:1n5 - methyl pentadecenoate; C16:0 - methyl hexadecanoate; C16:1n7 - methyl palmitoleate; C17:0 - methyl heptadecanoate; C17:1n7 - methyl heptadecenoate; C18:0 - methyl stearate; C18:1n9 - cis & trans-methyl oleate; C18:2n6 - cis-methyl linoleate; C18:2n6 - trans-methyl linoleate; C18:3n6 - methyl γ-linolenate; C18:3n3 - methyl α-linolenate; C20:0 - methyl eicosanoate; C20:1n9 - cis methyl 11-eicosatrienoate; C20:2n6 - cis-methyl 11,14-eicosadienoate; C21:0 - methyl heneicosanoate; C20:3n6 - cis-methyl 8,11,14,17-eicotrienoate; C20:3n3 - cis-methyl 11,14,17-eicotrienoate; C20:4n6 - methyl arachidonate; C20:5n3 - cis-methyl 5,8,11,14,17-eicosapentenoate; C22:0 - methyl behenate; C22:1n9 - methyl erucate; C22:2n6 - Methyl 13,16-docosadienoate; C22:6n3 - cis-methyl 4,7,10,13,16,19-docosahexenoate; C23:0 - methyl tricosanoate; C24:0 - methyl lignocerate; C24:1 - methyl nervonate.

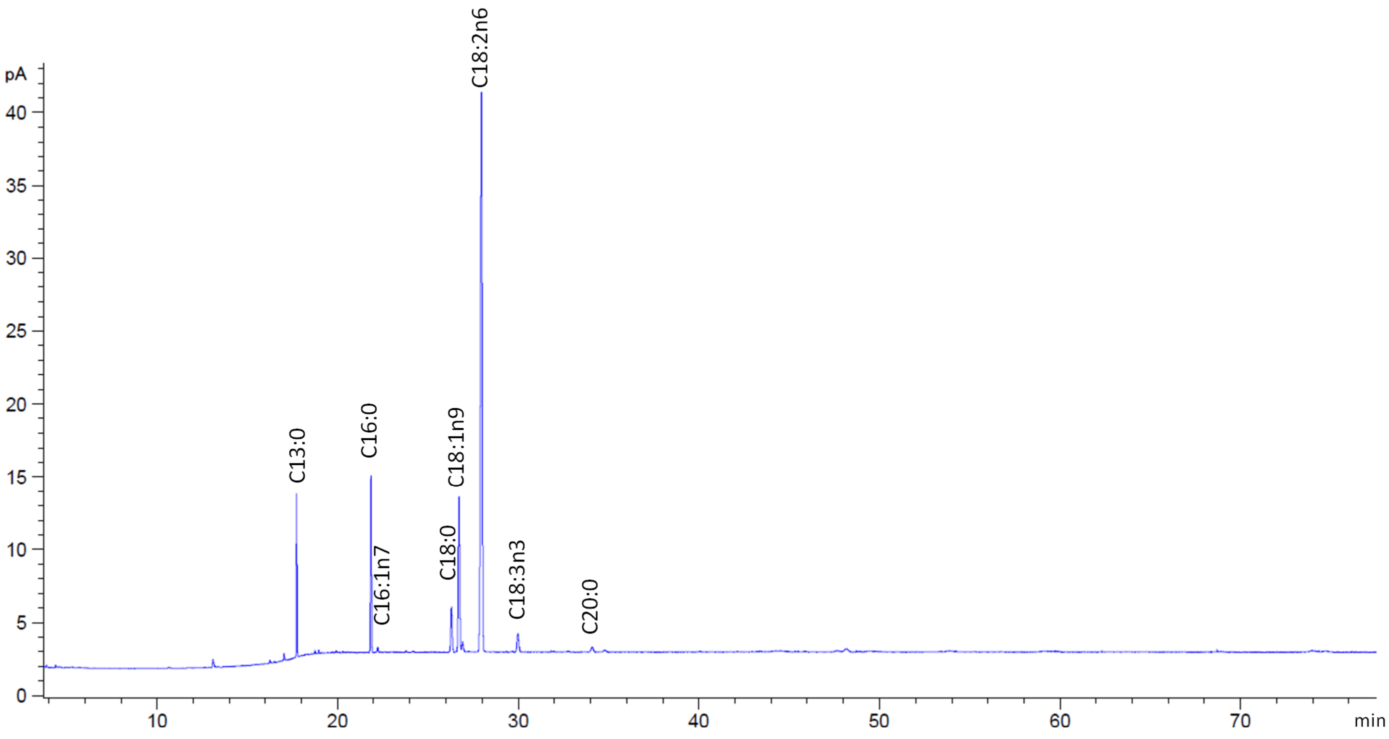

Figure S2. GC-FID chromatograms of fatty acids methyl esters (FAMEs) identified in grape pomace. Peak identification: C13:0 - methyl tridecanoate; C16:0 - methyl hexadecanoate; C16:1n7 - methyl palmitoleate; C18:0 - methyl stearate; C18:1n9 - cis & trans-methyl oleate; C18:2n6 - cis-methyl linoleate; C18:3n3 - methyl α-linolenate; C20:0 - methyl eicosanoate.

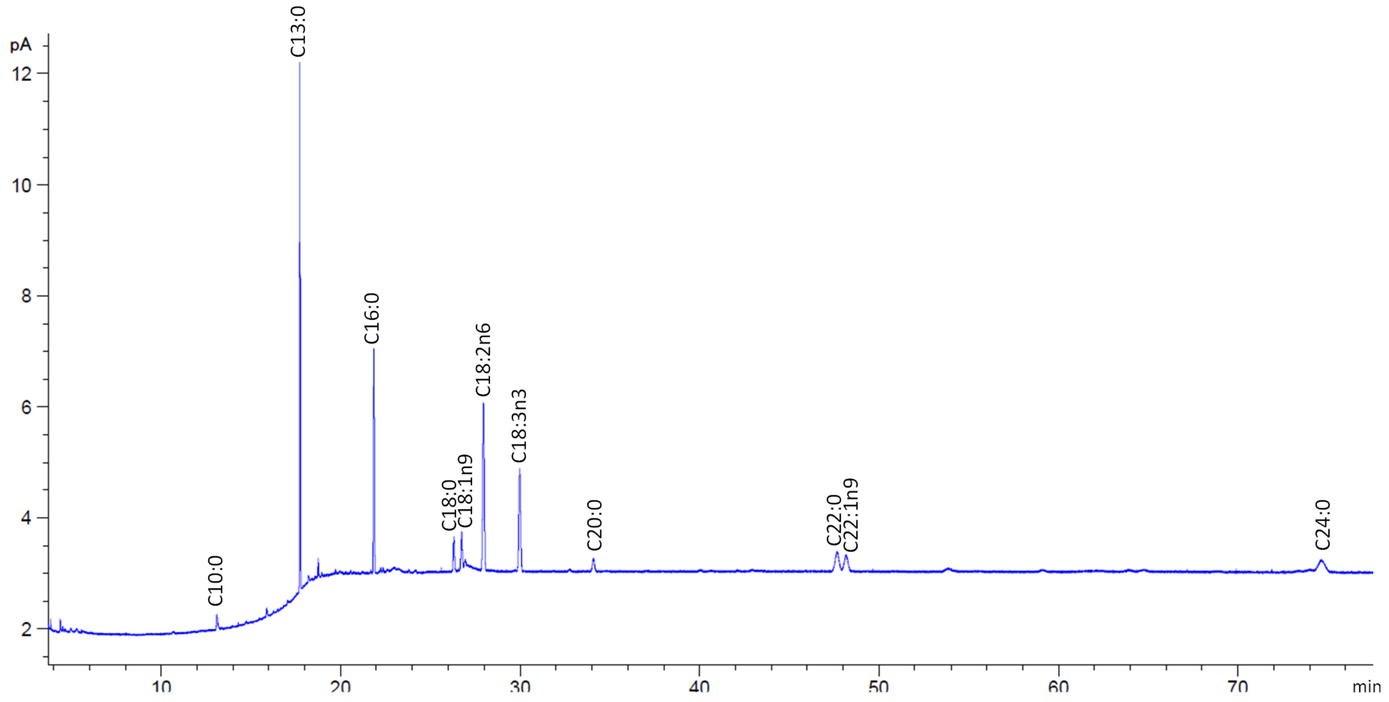

Figure S3. GC-FID chromatograms of fatty acids methyl esters (FAMEs) identified in grape stems. Peak identification: C10:0 - methyl decanoate; C13:0 - methyl tridecanoate; C16:0 - methyl hexadecanoate; C18:0 - methyl stearate; C18:1n9 - cis & trans-methyl oleate; C18:2n6 - cis-methyl linoleate; C18:3n3 - methyl α-linolenate; C20:0 - methyl eicosanoate; C22:0 - methyl behenate; C22:1n9 - methyl erucate; C24:0 - methyl lignocerate.

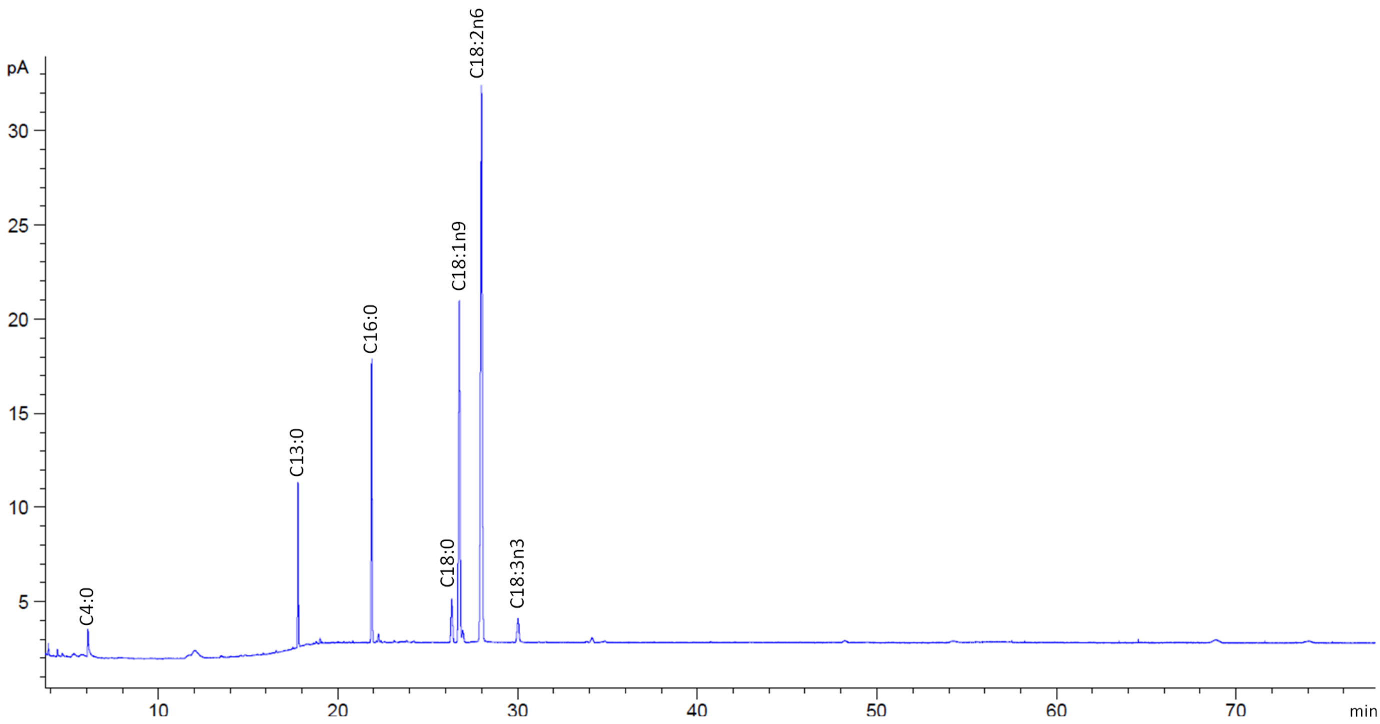

Figure S4. GC-FID chromatograms of fatty acids methyl esters (FAMEs) identified in grape bunches. Peak identification: C4:0 - methyl butanoate; C13:0 - methyl tridecanoate; C16:0 - methyl hexadecanoate; C18:0 - methyl stearate; C18:1n9 - cis & trans-methyl oleate; C18:2n6 - cis-methyl linoleate; C18:3n3 - methyl α-linolenate.

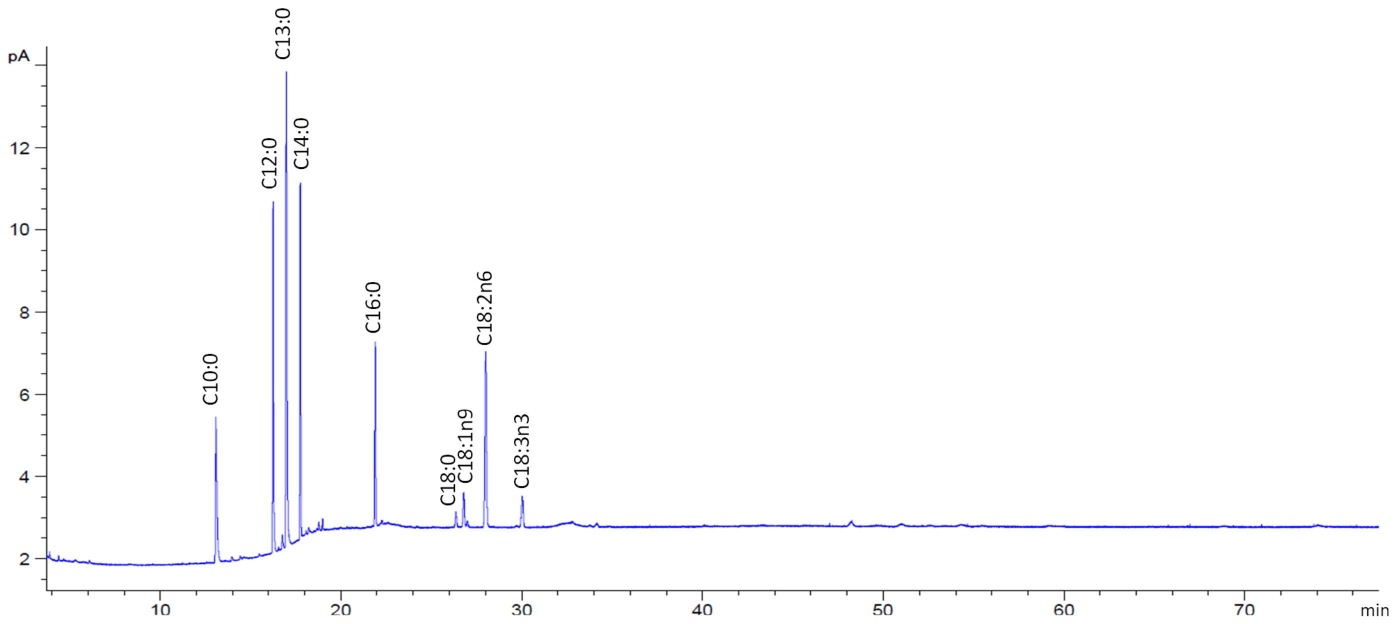

Figure S5. GC-FID chromatograms of fatty acids methyl esters (FAMEs) identified in white lees. Peak identification: C10:0 - methyl decanoate; C12:0 - methyl dodecanoate; C13:0 - methyl tridecanoate; C14:0 - methyl tetradecanoate; C16:0 - methyl hexadecanoate; C18:0 - methyl stearate; C18:1n9 - cis & trans-methyl oleate; C18:2n6 - cis-methyl linoleate; C18:3n3 - methyl α-linolenate.

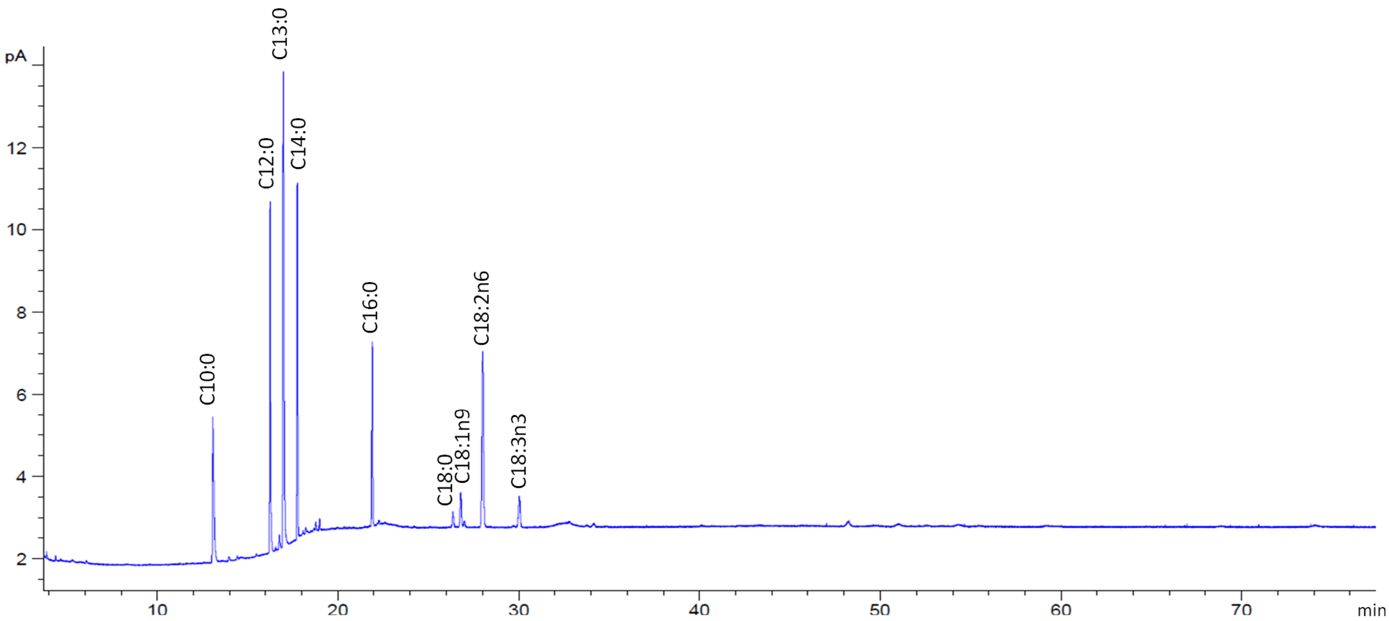

Figure S6. GC-FID chromatograms of fatty acids methyl esters (FAMEs) identified in red lees. Peak identification: C10:0 - methyl decanoate; C12:0 - methyl dodecanoate; C13:0 - methyl tridecanoate; C14:0 - methyl tetradecanoate; C14:1n5 - methyl 9-tetradecenoate; C16:0 - methyl hexadecanoate; C16:1n7 - methyl palmitoleate; C18:0 - methyl stearate; C18:1n9 - cis & trans-methyl oleate; C18:2n6 - cis-methyl linoleate; C18:3n3 - methyl α-linolenate.

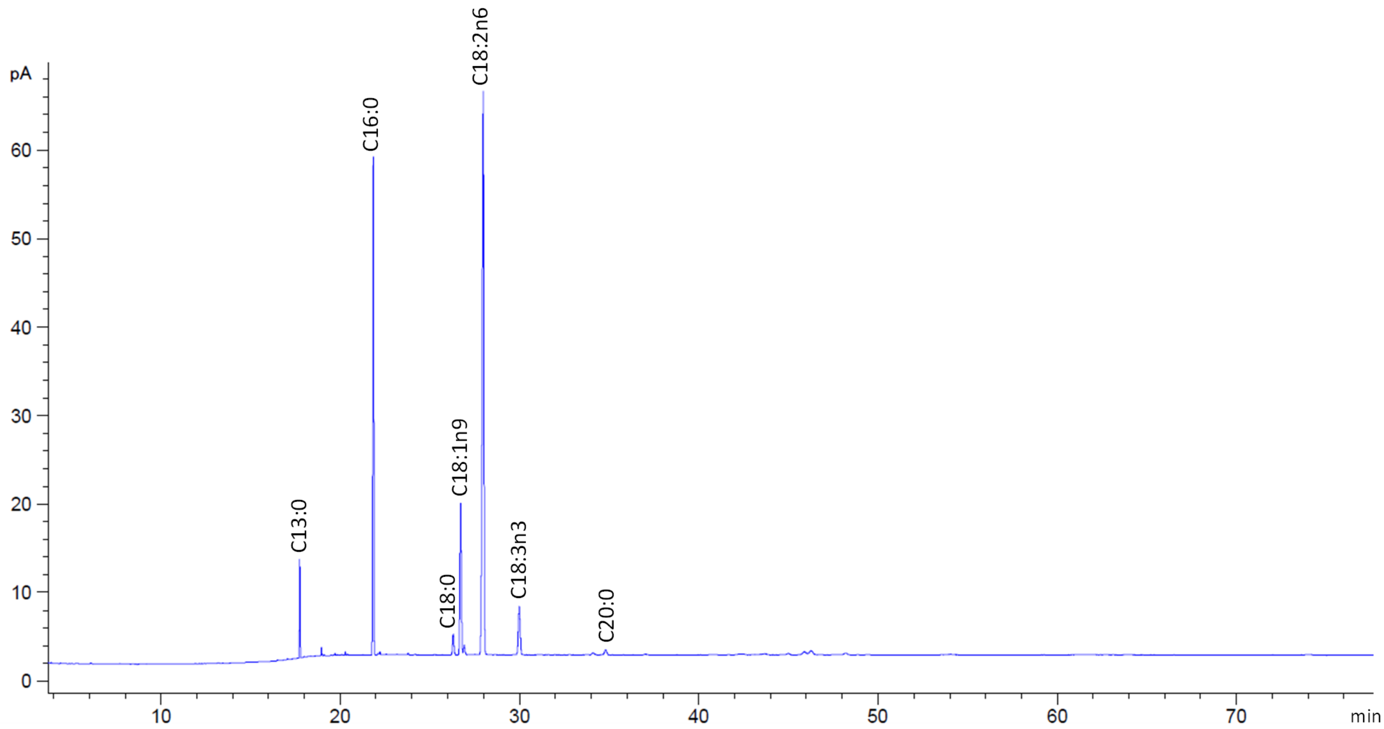

Figure S7. GC-FID chromatograms of fatty acids methyl esters (FAMEs) identified in BSG. Peak identification: C13:0 - methyl tridecanoate; C16:0 - methyl hexadecanoate; C18:0 - methyl stearate; C18:1n9 - cis & trans-methyl oleate; C18:2n6 - cis-methyl linoleate; C18:3n3 - methyl α-linolenate; C20:0 - methyl eicosanoate.

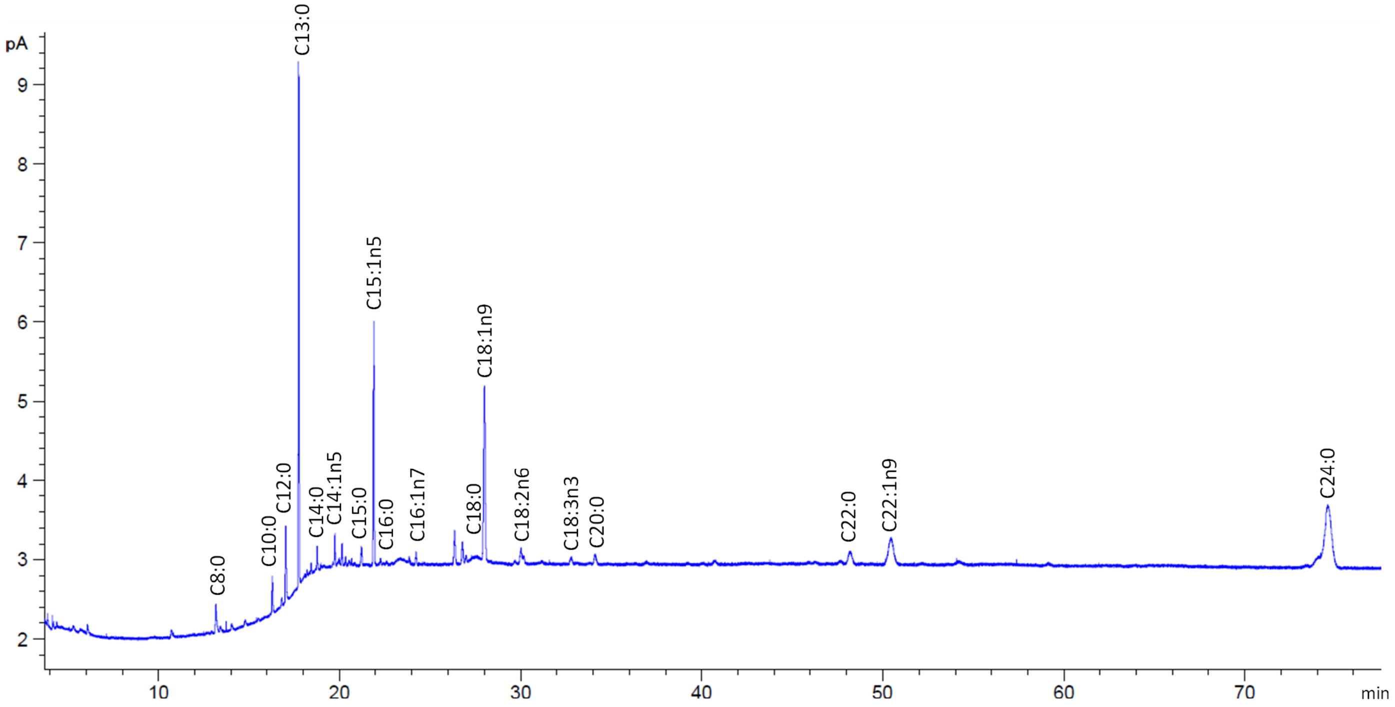

Figure S8. GC-FID chromatograms of fatty acids methyl esters (FAMEs) identified in carrot peel. Peak identification: C8:0 - methyl octanoate; C10:0 - methyl decanoate; C12:0 - methyl dodecanoate; C13:0 - methyl tridecanoate; C14:0 - methyl tetradecanoate; ; C14:1n5 - methyl 9-tetradecenoate; C15:0 - methyl pentadecanoate; C15:1n5 - methyl pentadecenoate; C16:0 - methyl hexadecanoate; C16:1n7 - methyl palmitoleate; C18:0 - methyl stearate; C18:1n9 - cis & trans-methyl oleate; C18:2n6 - cis-methyl linoleate; C18:3n3 - methyl α-linolenate; C20:0 - methyl eicosanoate; C22:0 - methyl behenate; C22:1n9 - methyl erucate; C24:0 - methyl lignocerate.

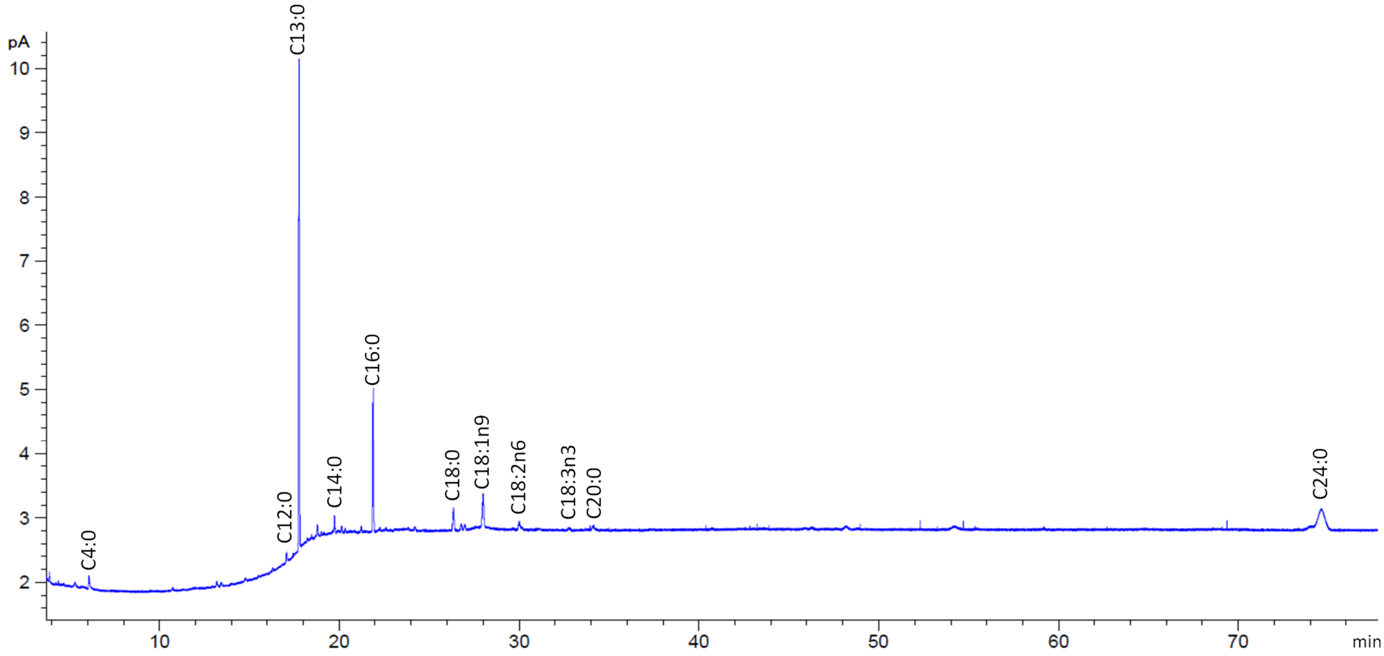

Figure S9. GC-FID chromatograms of fatty acids methyl esters (FAMEs) identified in potato peel. Peak identification: C4:0 - methyl butanoate; C12:0 - methyl dodecanoate; C13:0 - methyl tridecanoate; C14:0 - methyl tetradecanoate; C16:0 - methyl hexadecanoate; C18:0 - methyl stearate; C18:1n9 - cis & trans-methyl oleate; C18:2n6 - cis-methyl linoleate; C18:3n3 - methyl α-linolenate; C20:0 - methyl eicosanoate; C24:0 - methyl lignocerate.
